# Structural investigations on mechanism of lapatinib resistance caused by HER-2 mutants

**DOI:** 10.1371/journal.pone.0190942

**Published:** 2018-02-01

**Authors:** Sharad Verma, Sukriti Goyal, Anchala Kumari, Aditi Singh, Salma Jamal, Abhinav Grover

**Affiliations:** 1 School of Biotechnology, Jawaharlal Nehru University, New Delhi, India; 2 Department of Bioscience and Biotechnology, Banasthali University, Tonk, Rajasthan, India; 3 Department of Biotechnology, TERI School of Advanced Studies, Vasant Kunj, New Delhi, India; Universita degli Studi di Torino, ITALY

## Abstract

HER-2 belongs to the human epidermal growth factor receptor (HER) family. Via different signal transduction pathways, HER-2 regulates normal cell proliferation, survival, and differentiation. Recently, it was reported that MCF10A, BT474, and MDA-MB-231 cells bearing the HER2 K753E mutation were resistant to lapatinib. Present study revealed that HER-2 mutant K753E showed some contrasting behaviour as compared to wild, L768S and V773L HER-2 in complex with lapatinib while similar to previously known lapatinib resistant L755S HER-2 mutant. Lapatinib showed stable but reverse orientation in binding site of K753E and the highest binding energy among studied HER2-lapatinib complexes but slightly lesser than L755S mutant. Results indicate that K753E has similar profile as L755S mutant for lapatinib. The interacting residues were also found different from other three studied forms as revealed by free energy decomposition and ligplot analysis.

## Introduction

The epidermal growth factor receptor (EGFR), HER-2, HER-3, and HER-4 form the human epidermal growth factor receptor (HER) family. Via different signal transduction pathways. HER family regulates normal cell survival, proliferation and differentiation [1]. The intracellular domain of HER-2 is composed of three parts which contains ~ 500 residues, namely a cytoplasmic juxta membrane linker, a tyrosine kinase (TK) domain and a carboxyl-terminal tail [2, 3].

The contribution of HER-2 mutations is 1–4% in lung cancer, 1.67% in breast cancer, and 2.9% in colorectal [4–11]. Among all cases of invasive breast cancer, approximately 20–25% is HER-2-positive breast cancer. These are accompanying higher risk for progression, and reduced overall survival [12, 13]. While drugs such as trastuzumab and lapatinib have revealed significant effectiveness in treating HER-2-positive breast cancer patients [14–18], progressive drug resistance in HER-2-positive breast cancer patients has also been witnessed [19, 20].

In a recent study on HER-2-negative tumors, novel mutations L768S and V773L were detected, whereas in HER2-positive another novel mutation K753E was found. The cells bearing the mutations L768S and V773L exhibited more rapid growth while cells bearing the K753E mutation were resistant to lapatinib [21]. Extensive research has been conducted regarding the molecular mechanisms leading to trastuzumab and lapatinib resistance [18], including the overexpression or hyper expression of other HER family receptors and their ligands [21].

In recent years, molecular dynamics (MD) simulations based studies have demonstrated great potential as a substitute for investigations in understanding the dynamical aspects of protein-ligand interaction, protein folding, and also protein-protein interaction [22–30]. In the present study, to gain insights into the effects of mutations (K753E, L768S and V773L) on the binding strength of lapatinib, MD simulations of the kinase domain of HER-2 (wild and mutants) in complex with the lapatinib were performed. We quantitatively evaluated the structural differences in HER-2 mutants with respect to initial structures using the root mean square deviation (RMSD) of the backbone of a protein. RMSD of lapatinib was also calculated in binding with all HER-2 mutants. To study change in molecular size over MD simulation time, the gyration radius (Rg) of the studied HER-2 mutants in lapatinib binding state was calculated. The Rg value represents the mean squared distance of the atoms from the centre of mass of the molecule [31]. To determine which parts of HER-2 mutants in lapatinib binding state are influenced by lapatinib, we compared the fluctuation of residues of studied HER-2 mutants in the binding state. We also calculated the free energy between HER-2 forms and lapatinib which provides valuable insights into the recognition and binding of the drug and elucidate the effect of these mutations on drug recognition and binding. To further explore the conformational space of HER-2 mutants stabilized by the lapatinib, the probability-based free energy landscapes (FEL) as a function of the first two principal components (PC1 and PC2) were relatively investigated.

## Materials and methods

### Molecular dynamics simulations

Classical MD simulations were performed for HER-2 wild and mutant forms with lapatinib using the GROMACS package [32, 33]. The initial structure of HER-2 was taken from PDB ID:3PP0. Discovery studio was used to generate HER-2 mutants (Discovery studio client). To generate the topologies for proteins and for the simulations, the Gromos43a1 force field [34] was used. Energy minimization, position restrain dynamics and final production MD simulations for 50 ns were carried out as described in our previous research papers [25, 35]. Chimera was used to visualize the complexes [36].

### Binding free energy calculations

Binding free energy calculations were performed from the snapshots of MD trajectory using the molecular mechanics Poisson Boltzmann surface area (MM/PBSA) method [37]. The binding free energy of the HER-2-lapatinib complexes were analyzed by taking snapshot at an interval of 1.5 ps from 45 to 50 ns MD simulation during equilibrium phase, using g_mmpbsa tool of GROMACS [38].

### Free energy landscape (FEL)

The backbone atoms of HER-2 were selected to perform PCA in each system. PCA elucidates the essential motions which lead conformational shifts during the simulations [39–42]. The description of sub conformational changes in protein is provided by only the first few eigenvectors which represent the most principal collective motions [43]. The GROMACS in-built tool, gmx anaeig, was used to analyze and plot the PC1 and PC2 [44]. The g_sham module of GROMACS package was used to analyse the 2D representation of the FEL calculating the free energy (G) by the first two eigenvectors extracted after PCA.

## Results and discussion

**Fig 1:**
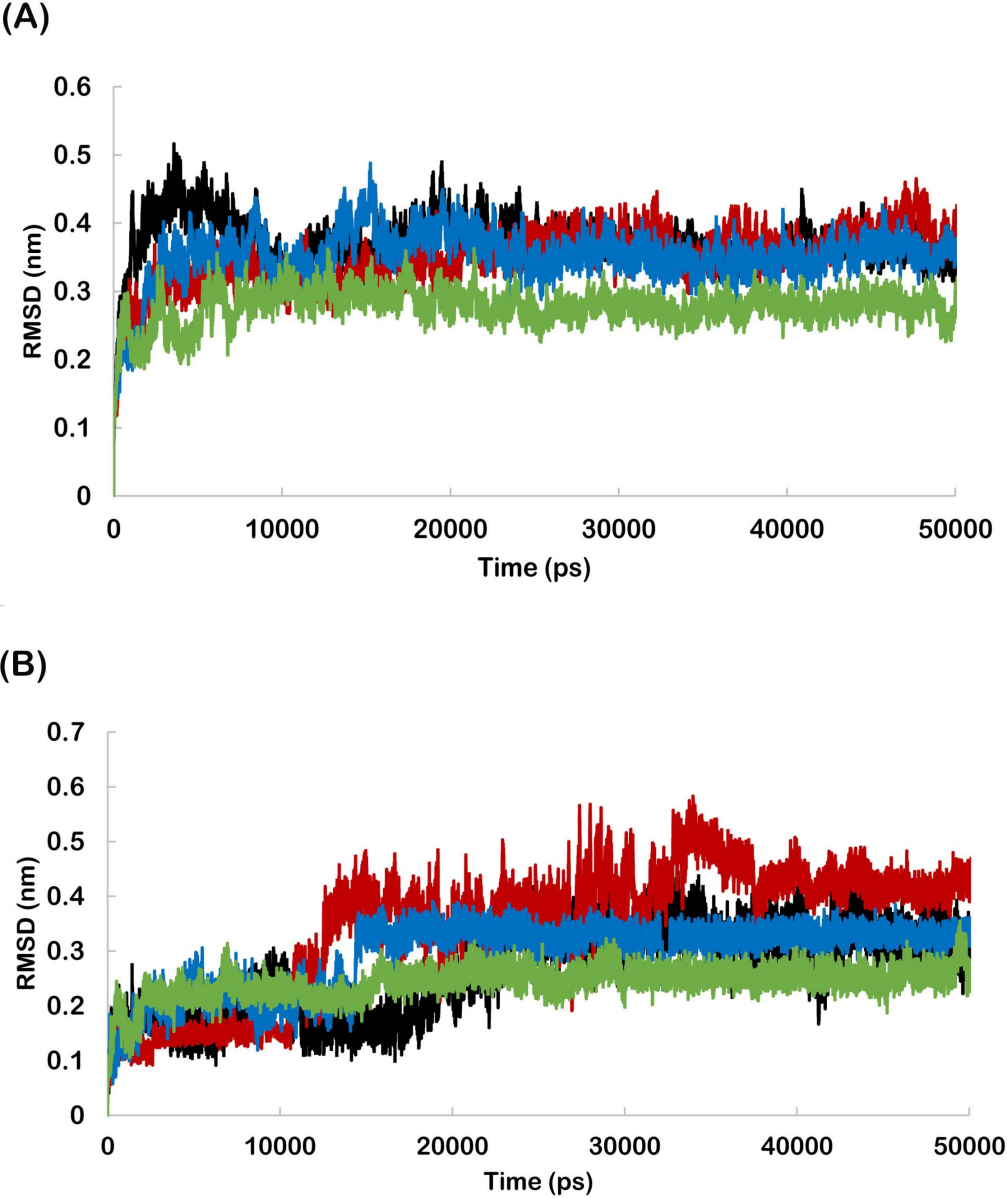
(A) Backbone RMSD’s as a function of time for wild and mutant HER-2 in complex with lapatinib, (B) RMSD’s of lapatinib in wild and mutant HER-2 binding site. (Black-wild, Red- K753E, Blue-L768S, Green -V773L).

**Fig 2:**
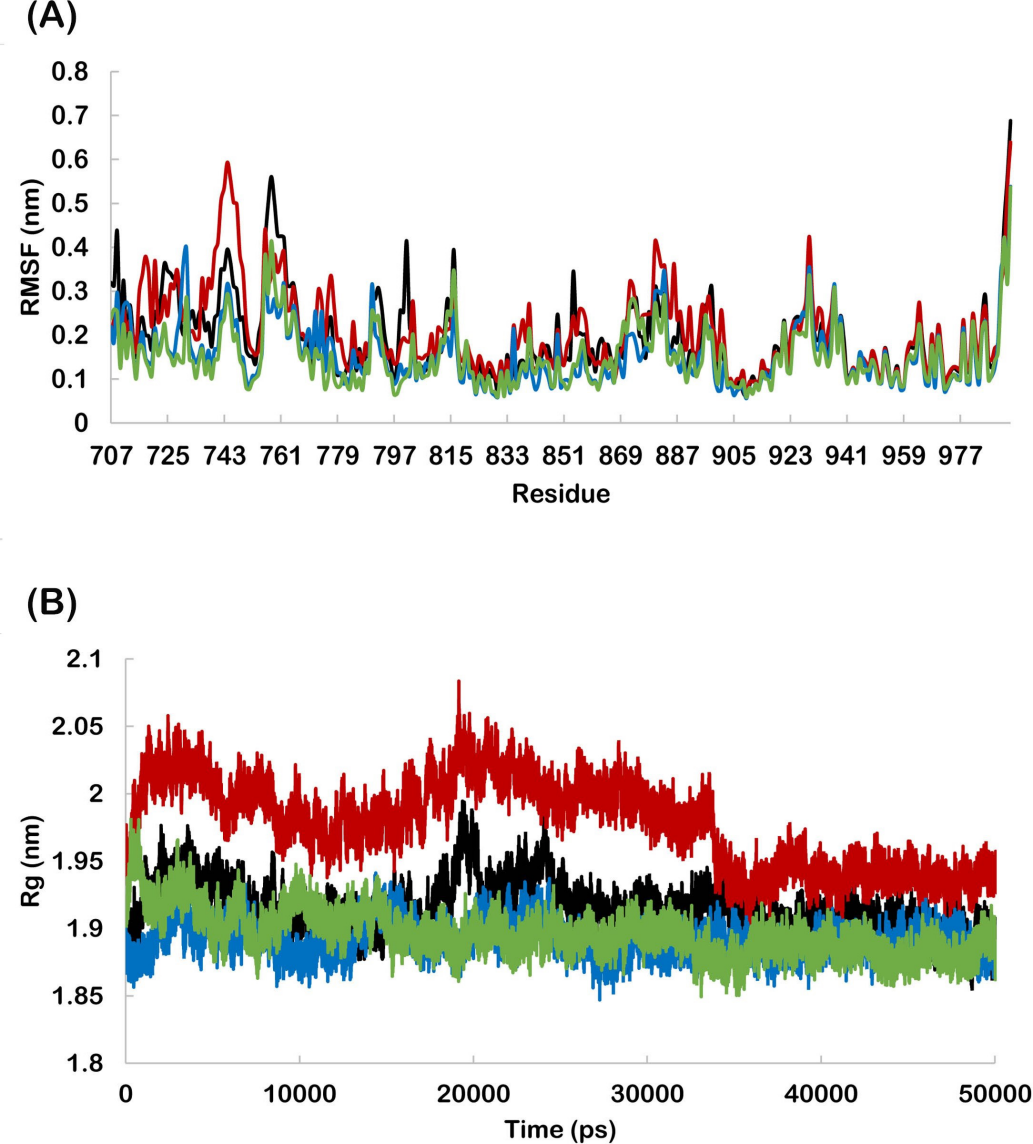
(A) RMSF profile of wild and mutant HER-2 residues in complex with lapatinib. (B) Radius of gyration of backbone of wild and mutant HER-2 in complex with lapatinib. (Black-wild, Red- K753E, Blue-L768S, Green -V773L).

The RMSD profiles were found to be below 0.50 nm for wild and mutant HER-2 backbone during the entire simulation period indicating the suitability of all systems for further analyses (Fig 1A). The RMSD of lapatinib bound to wild and mutant HER-2 was also calculated to analyse the fluctuation from initial position. The lapatinib bound to K753E showed the highest RMSD. In case of wild and L768S, RMSD of lapatinib was found very close to each other. V773L bound lapatinib showed the lowest RMSD (Fig 1B). This revealed the highest stability of lapatinib in V773L binding site in initial orientation. RMSF profile revealed that the residue fluctuation in K753E was more as compared to other forms (Fig 2A) and similarly, K753E has higher radius of gyration (Fig 2B).

### Free energy calculations

**Fig 3:**
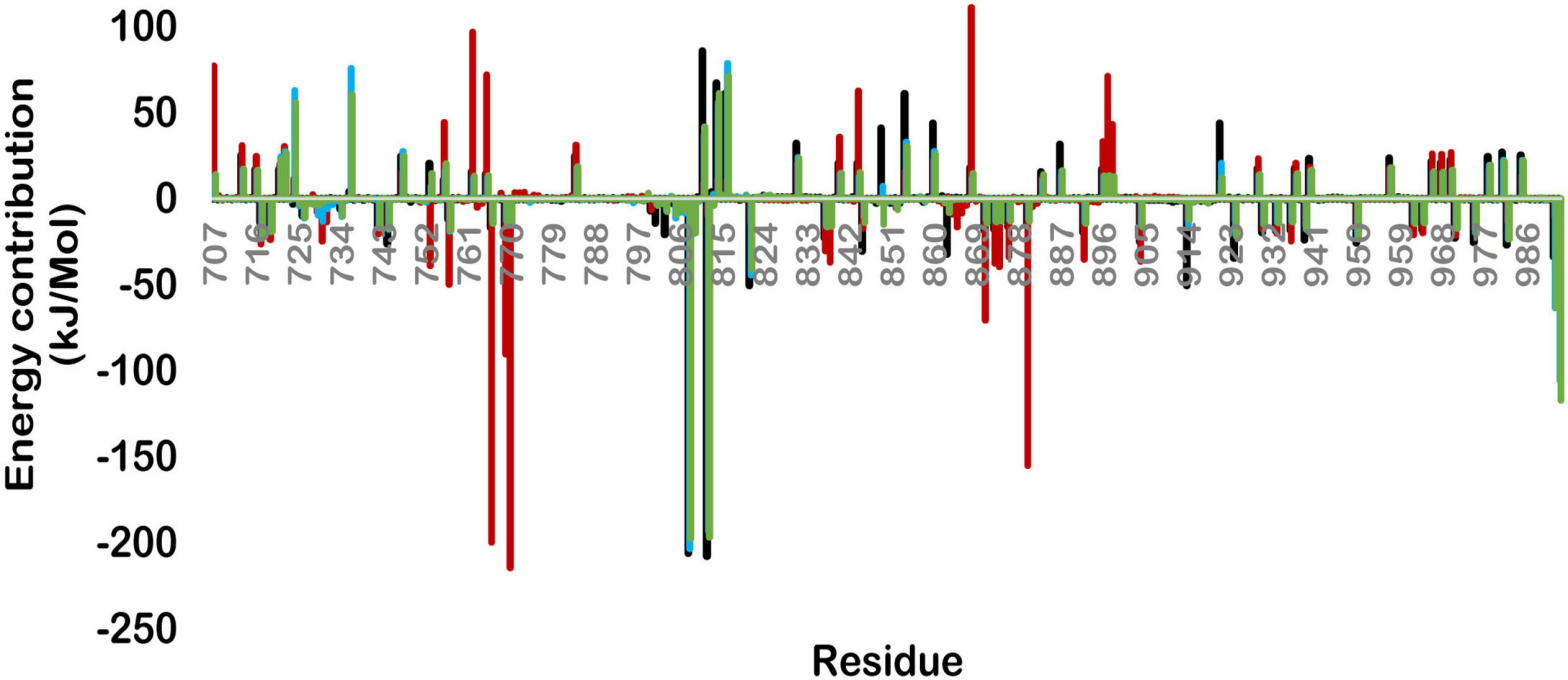
Free energy decomposition showing the contribution of residues in term of binding energy for wild and mutant HER-2-lapatinib complexes (Black-wild, Red- K753E, Blue-L768S, Green -V773L).

The MM/PBSA calculations were performed for all the HER2-lapatinib complexes and binding free energy components (Table 1). All mutants were found to be at highly favourable state than the wild type for lapatinib. The final binding energy for wild, K753E, L768S and V773L were -554.48, -811.89, -600.80 and -668.68 kJ/mol respectively. The electrostatic energy contribution in MM energy was found highest in case of K753E among the four forms. All mutant forms were found to have more vdW interactions as compared to wild (Table 1). These results indicate that studied mutations in HER2 increased the MM energy for lapatinib which is the most significant term in calculation of binding energy. To determine the contribution of each HER-2 residue which is involved in any type of interaction with inhibitor, we performed free energy decomposition and calculated per residues binding energy (Fig 3). The K753E form of HER-2 was found to have a very different profile of contributing residues as compared to rest of other three forms. The major contributing residues were Glu766, Asp769, Glu770, Asp871, and Asp880. These residues were not involved in interaction with lapatinib in other HER-2 forms. In case of wild, L768S and V773L residues Asp808 and Glu812 were found to be the most significant contributor and were found absent in K753E. These results revealed that lapatinib binding orientation in K753E is different from rest of three forms.

**Table 1:** MM/PBSA free energy calculation of wild and mutant HER-2-Lapatinib complexes.

HER-2 form	MM energy (kJ/mol)		Polar solvation (kJ/mol)	SASA (kJ/mol)	Binding energy (kJ/mol)
	vdW	Electrostatic			
**wild**	-130.00	-1442.98	1037.83	-19.32	-554.48
**K753E**	-233.98	-1777.05	1209.19	-10.05	-811.89
**L768S**	-259.61	-1395.93	1070.36	-15.62	-600.80
**V773L**	-194.02	-1581.82	1131.67	-23.84	-668.68

**Fig 4:**
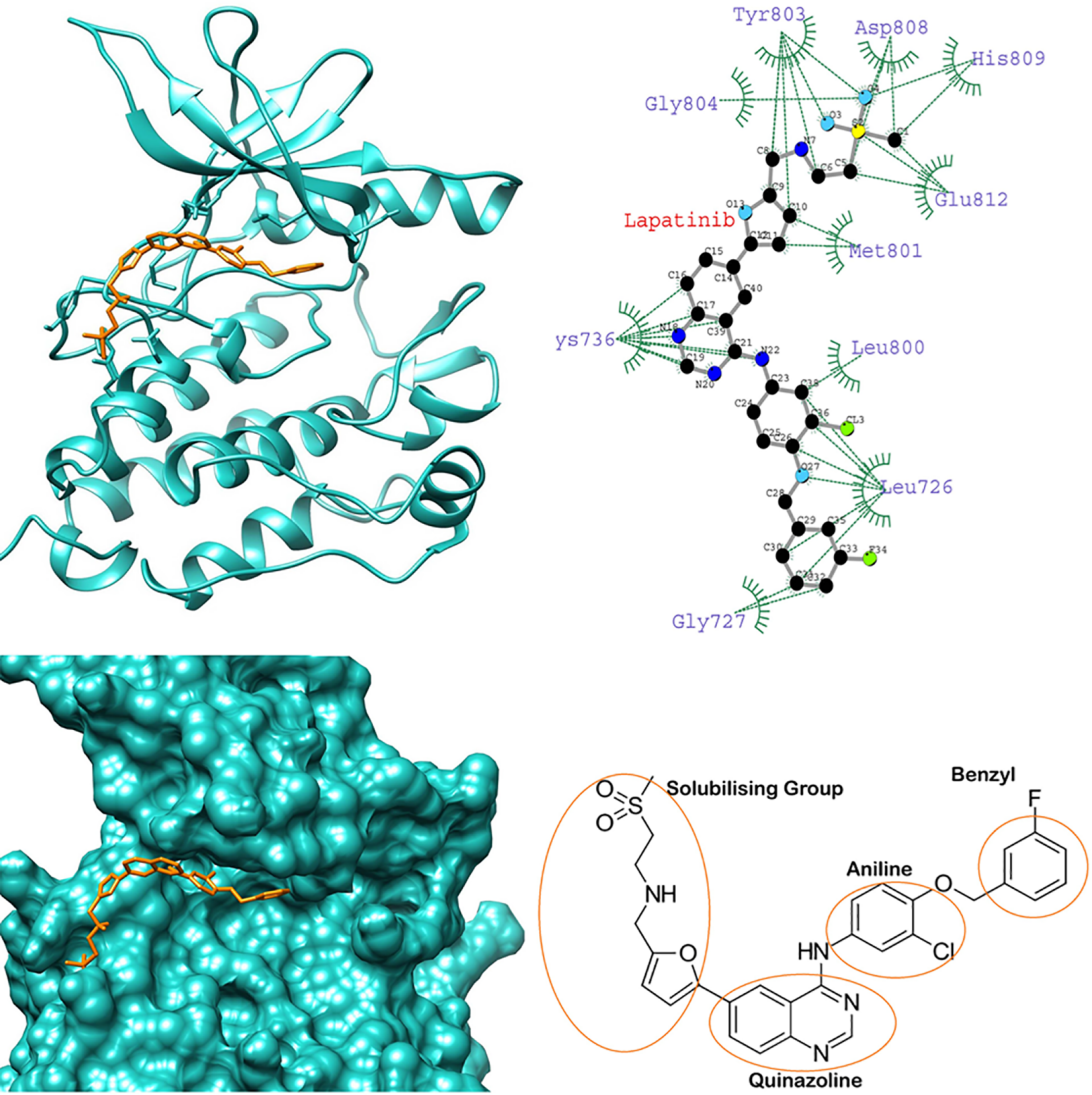
Average structure of wild HER-2-lapatinib complex (left top), surface representation of wild HER-2 binding site with lapatinib (left bottom), Interaction plot of wild HER-2-lapatinib using average structure by LIGPLOT (right top) (Dotted green and dotted red lines represent hydrophobic contacts and hydrogen bonds respectively), chemical structure of lapatinib (right bottom).

**Fig 5:**
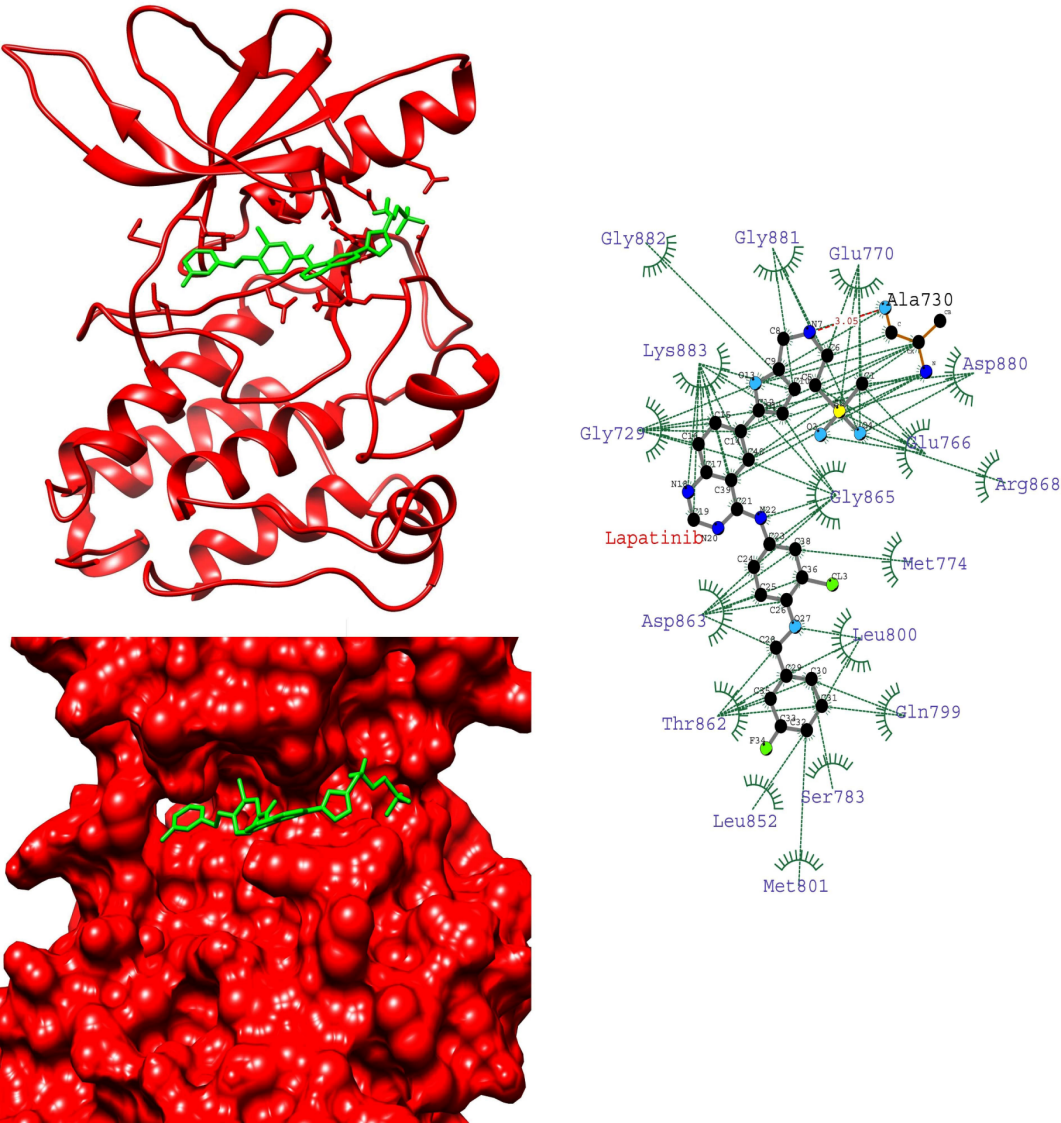
Average structure of K753E HER-2-lapatinib complex (left top), surface representation of K753E HER2 binding site with lapatinib (left bottom), Interaction plot of K753E HER-2-lapatinib using average structure by LIGPLOT (right) (Dotted green and dotted red lines represent hydrophobic contacts and hydrogen bonds respectively).

**Fig 6:**
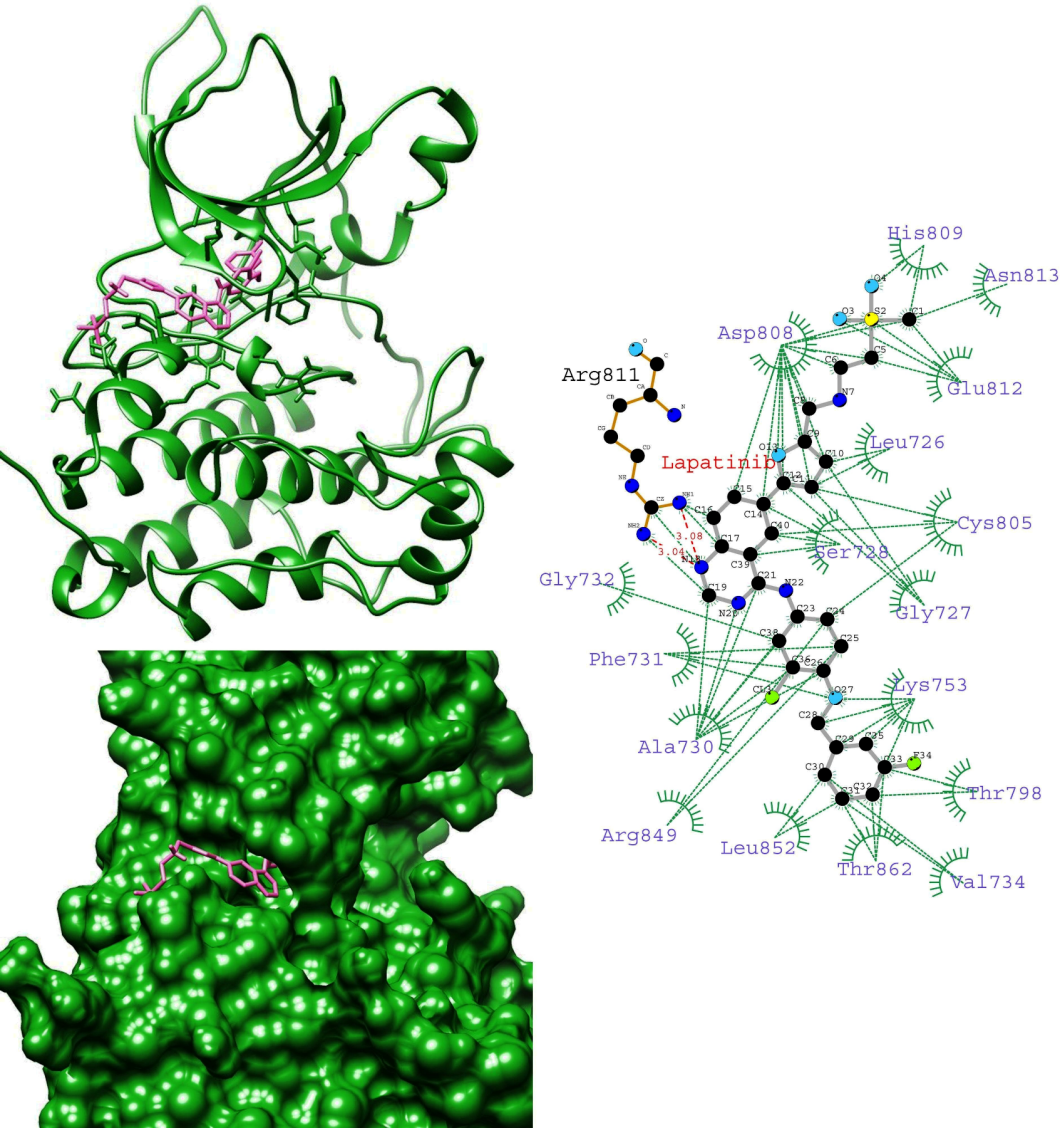
Average structure of L768S HER-2-lapatinib complex (left top), surface representation of L768S HER-2 binding site with lapatinib (left bottom), Interaction plot of L768S HER-2-lapatinib using average structure by LIGPLOT (right) (Dotted green and dotted red lines represent hydrophobic contacts and hydrogen bonds respectively).

**Fig 7:**
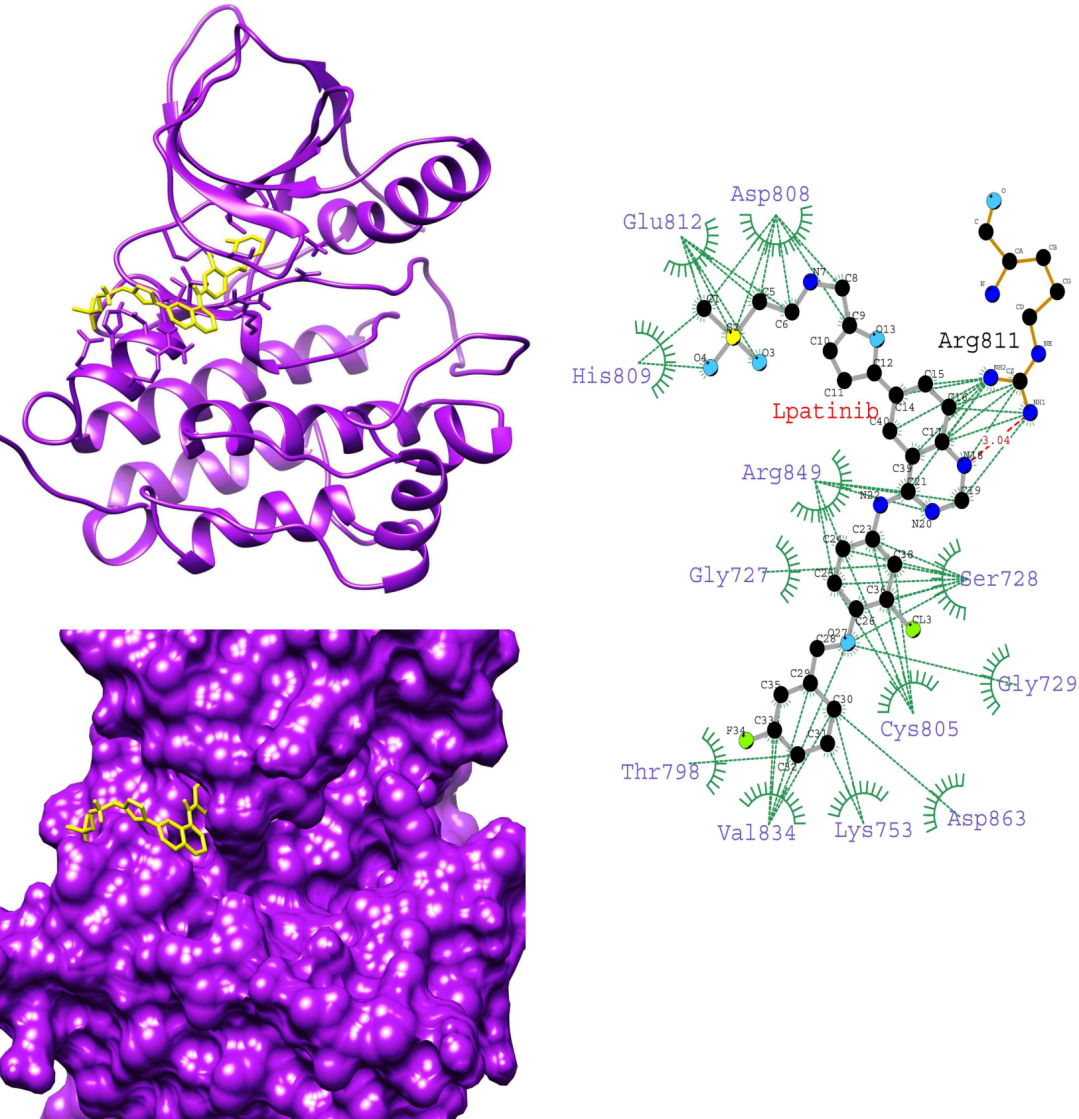
Average structure of V773L HER-2-lapatinib complex (left top), surface representation of V773L HER-2 binding site with lapatinib (left bottom), Interaction plot of V773L HER-2-lapatinib using average structure by LIGPLOT (right) (Dotted green and dotted red lines represent hydrophobic contacts and hydrogen bonds respectively).

To gain insight into the average binding pose of lapatinib, we extracted the average structure of HER-2-lapatinib complexes. We found that the orientation of lapatinib in K753E is reverse in the binding site while compared with other complexes (Figs 4–7). This suggests that in K753E, lapatinib binding is energetically more favourable in reverse orientation which leads to more binding energy. Ligplot analysis of average structure revealed that the solubilizing group interacts with residues Asp808, His809 and Glu812 were common residues in wild, L768S and V773L forms while in case of K753E Glu766, Asp880 and Arg868 were interacting residues (Figs 4–7). These results indicate the reverse orientation of lapatinib in K753E stabilized the complex in more favourable manner.

### Free energy landscape (FEL)

**Fig 8:**
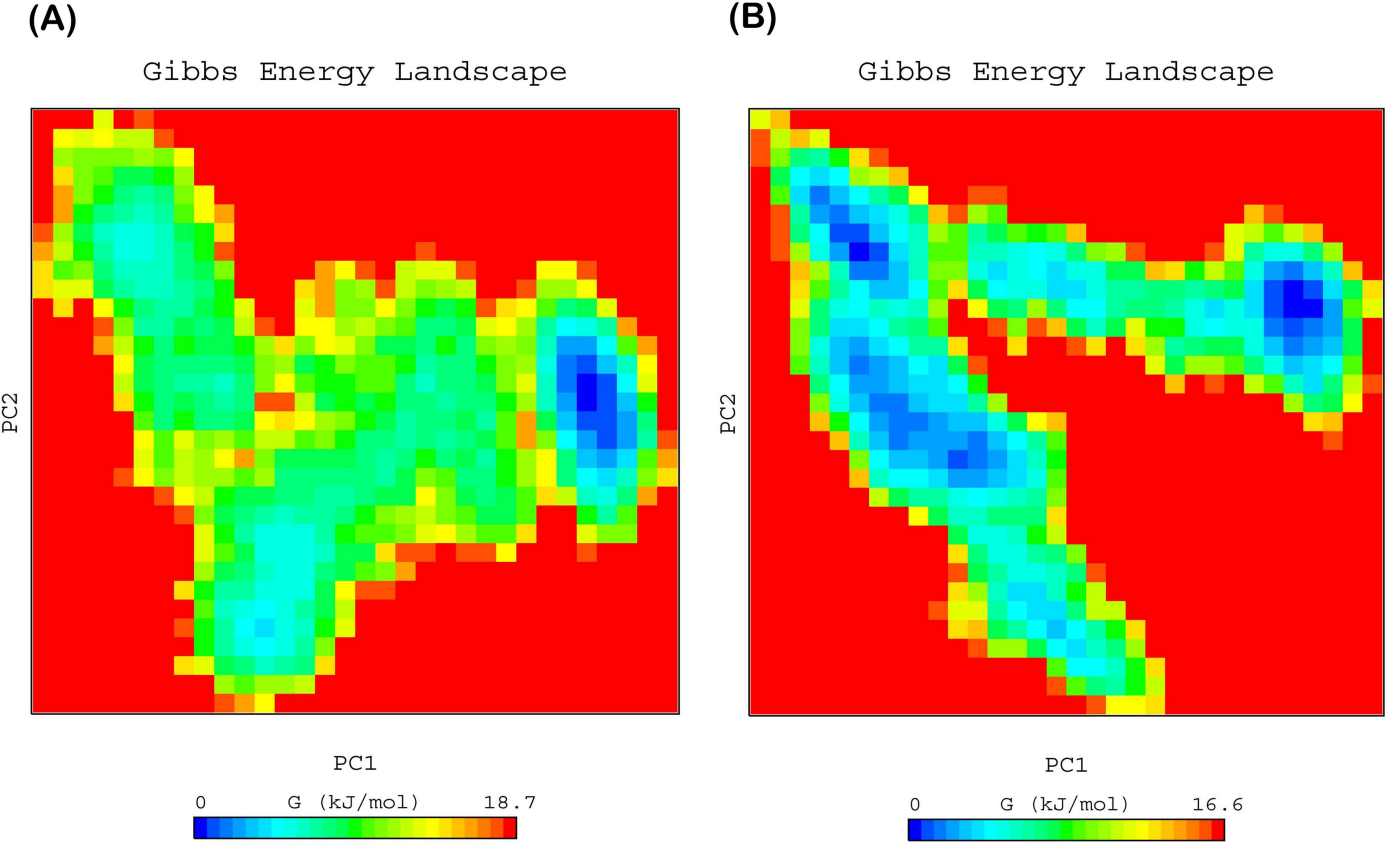
Free energy landscape of HER-2 as a function of first two principal components PC1 and PC2 (A) wild, (B) K753E.

**Fig 9:**
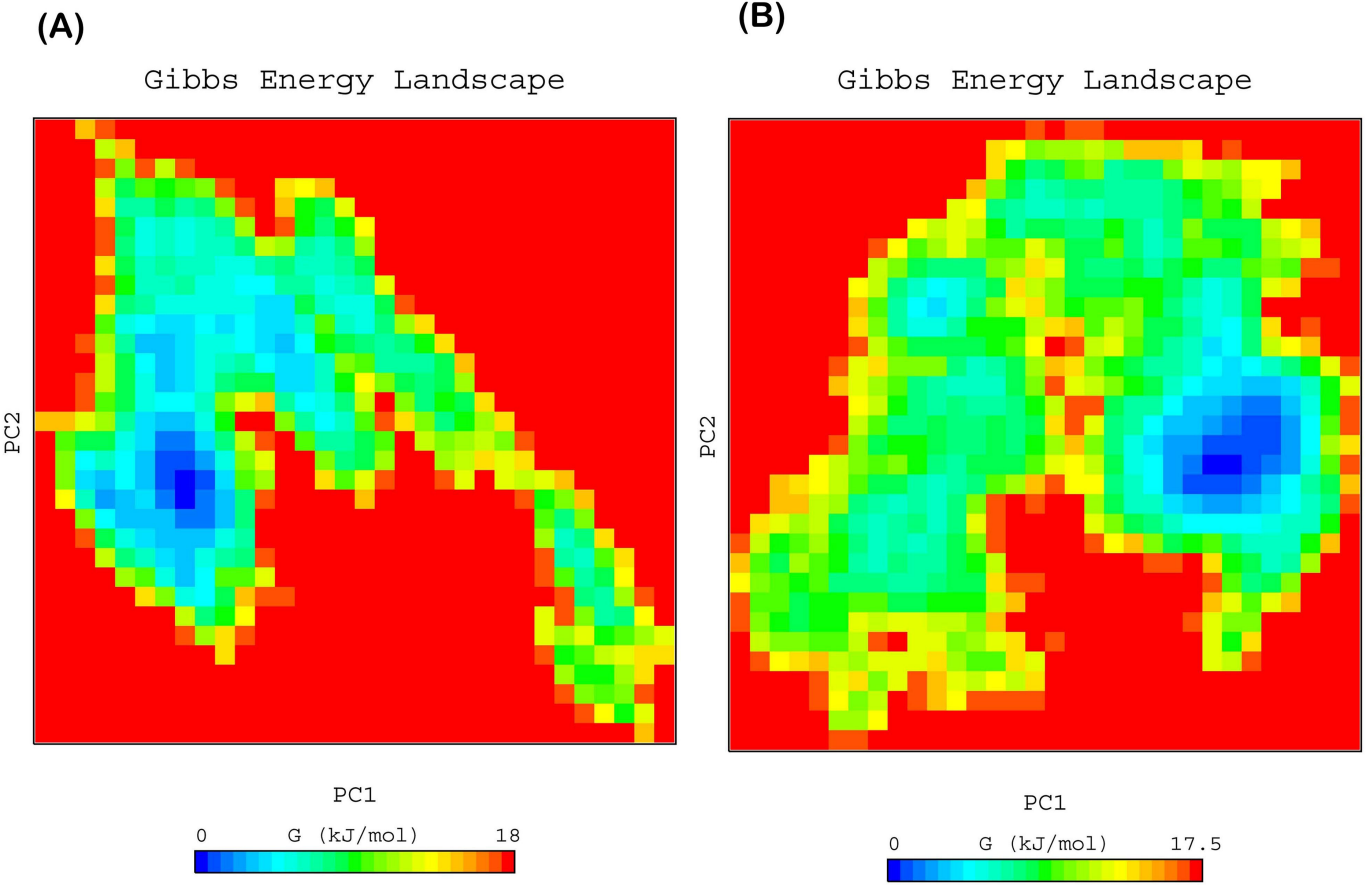
Free energy landscape of HER-2 as a function of first two principal components PC1 and PC2 (A) L768S, (B) V773L.

To elucidate the sub conformational patterns of HER-2 forms and lapatinib complexes, we studied the FEL against first two principal components PC1 and PC2 which revealed ΔG value 0 to 18.70, 16.60 (Fig 8), 18.0 and 17.5 kJ/mol (Fig 9) for wild, K753E, L768S and V773L respectively. The size and shape of the minimal energy area (in blue) reveals the stability of a complex. Smaller and more concentrated blue areas suggest more stability of the corresponding complex [45]. All the HER-2 forms showed more centralized and concentrated minima except K753E. K753E HER-2 showed several minima which indicate that protein-lapatinib complex switch to various conformations to achieve minima. This is further support the change in orientation of lapatinib in binding site.

**Fig 10:**
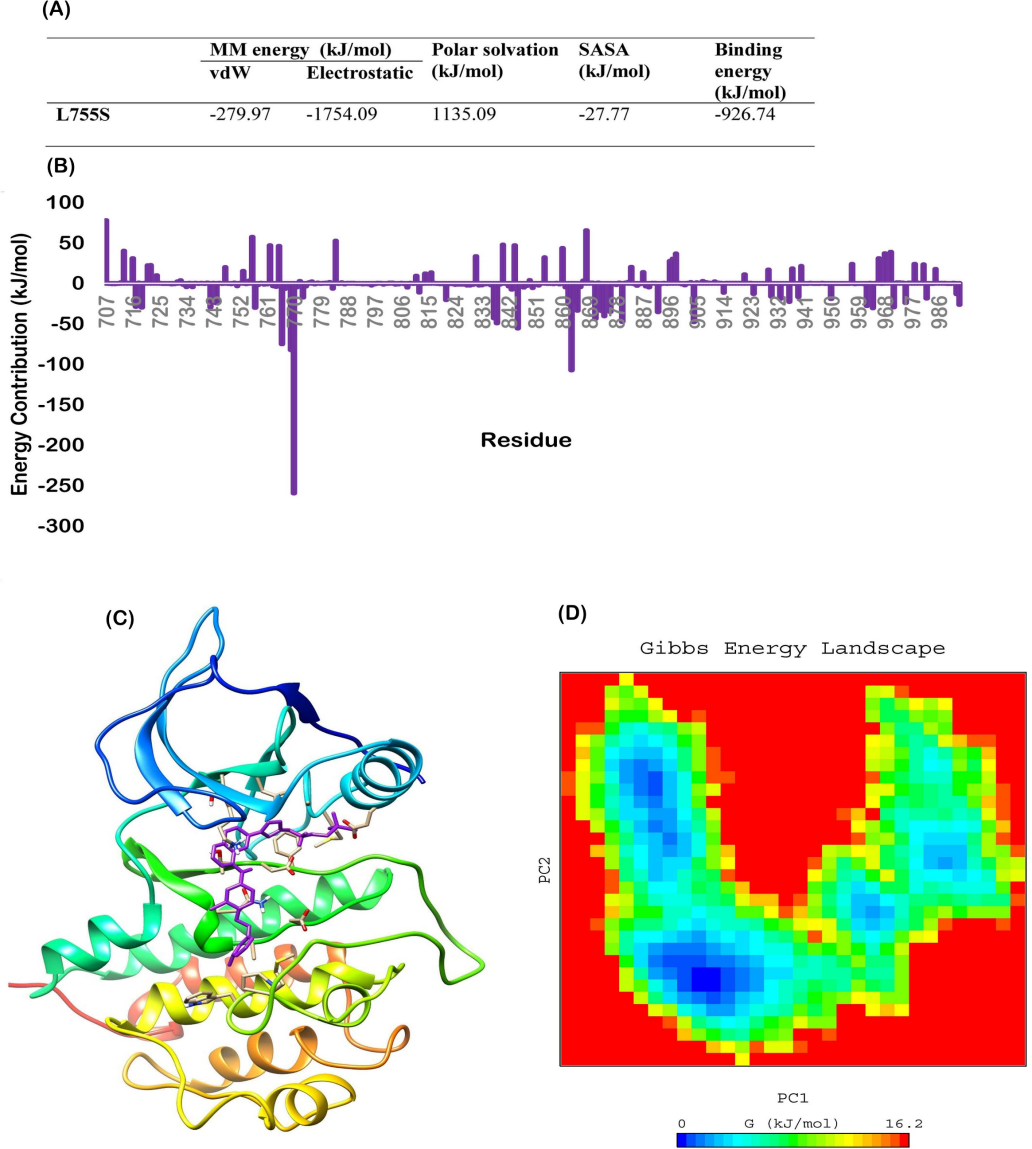
(A) MM/PBSA free energy calculation of HER-2 L755S-Lapatinib complex, (B) Free energy decomposition showing the contribution of residues in term of binding energy for HER-2 L755S-lapatinib complex (C) Average structure of HER-2 L755S-lapatinib complex, (D) Free energy landscape of HER-2 L755S.

The cells bearing K753E mutation were found to be resistant to lapatinib. However the reason of resistance may be the overexpression of mutant HER-2. Same study have shown that the phosphorylation of downstream signalling protein PCLγ decreased as the concentration of lapatinib increased in MCF10A cell lines [21]. In previous studies it was reported that L755S mutation bearing cells showed resistance towards lapatinib might be due to overexpression of protein [46–48]. To compare with the L755S, we performed molecular dynamics simulation of HER-2 L755S-lapatinib complex and MM/PBSA calculations. Binding energy of HER-2 L755S-lapatinib complex was found -926.74 kJ/mol (Fig 10A) which is the highest in all studied complexes and close to K753E. Further free energy decomposition revealed that the contributing residues profile is very similar to the K753E. Glu766, Asp769, Glu770 and Asp871were the foremost contributing residues which were interacted with lapatinib (Fig 10B). Average structure revealed the orientation of lapatinib is similar to K753E (Fig 10C). The FEL analysis of L755S complex showed several minima as found in case of K753E (Fig 10D). Endogenous expression of L755S mutant HER-2 in models that do not have overexpression not sufficient to produce resistance to lapatinib due to lower levels of the mutant protein and show sensitivity towards lapatinib [49]. Zuo et al. [21] confirmed that the HER-2 K753E mutation may induce lapatinib resistance due to its proximity to the lapatinib-resistant mutation L755S and cell lines with the HER-2 K753E mutation overexpression were certainly resistant to lapatinib.

Our findings show consistency with the previous cell line studies and indicates that K753E lapatinib drug resistant mutant have more affinity towards lapatinib as compared to wild and other mutants so the loss of interaction might not be the reason behind the lapatinib resistance. Overexpression of mutant K753E HER-2 might be the cause of lapatinib resistance as found in case of L755S.

## Conclusion

Present study revealed that mutant K753E was found to have some contrasting behaviour as compared to wild, L768S and V773L. Lapatinib showed stable reverse orientation in binding site of K753E which may be reason behind the highest binding energy among studied HER-2-lapatinib complexes but slightly lesser than L755S mutant. The interacting residues were also found different from other three studied forms as revealed by free energy decomposition and ligplot analysis. Results indicate that K753E has similar profile as L755S mutant for lapatinib. Overexpression of mutant K753E HER-2 might be the cause of lapatinib resistance as found in case of L755S.

## References

[1] Iqbal N, Iqbal N. Human epidermal growth factor receptor 2 (HER2) in cancers: overexpression and therapeutic implications. Molecular biology international. 2014;2014. 10.1155/2014/852748 PMC4170925 25276427

[2] Telesco SE, Radhakrishnan R. Atomistic insights into regulatory mechanisms of the HER2 tyrosine kinase domain: a molecular dynamics study. Biophysical journal. 2009;96(6):2321–34. doi: 10.1016/j.bpj.2008.12.3912 19289058 10.1016/j.bpj.2008.12.3912 PMC2907720

[3] Lemmon MA, Schlessinger J. Cell signaling by receptor tyrosine kinases. Cell. 2010;141(7):1117–34. doi: 10.1016/j.cell.2010.06.011 20602996 10.1016/j.cell.2010.06.011 PMC2914105

[4] Yan M, Parker BA, Schwab R, Kurzrock R. HER2 aberrations in cancer: implications for therapy. Cancer treatment reviews. 2014;40(6):770–80. doi: 10.1016/j.ctrv.2014.02.008 24656976 10.1016/j.ctrv.2014.02.008

[5] Lee JW, Soung YH, Seo SH, Kim SY, Park CH, Wang YP, et al Somatic mutations of ERBB2 kinase domain in gastric, colorectal, and breast carcinomas. Clinical cancer research. 2006;12(1):57–61. doi: 10.1158/1078-0432.CCR-05-0976 16397024 10.1158/1078-0432.CCR-05-0976

[6] Sasaki H, Shimizu S, Endo K, Takada M, Kawahara M, Tanaka H, et al EGFR and erbB2 mutation status in Japanese lung cancer patients. International journal of cancer. 2006;118(1):180–4. doi: 10.1002/ijc.21301 16003726 10.1002/ijc.21301

[7] Ding L, Getz G, Wheeler DA, Mardis ER, McLellan MD, Cibulskis K, et al Somatic mutations affect key pathways in lung adenocarcinoma. Nature. 2008;455(7216):1069–75. doi: 10.1038/nature07423 18948947 10.1038/nature07423 PMC2694412

[8] Minami Y, Shimamura T, Shah K, LaFramboise T, Glatt KA, Liniker E, et al The major lung cancer-derived mutants of ERBB2 are oncogenic and are associated with sensitivity to the irreversible EGFR/ERBB2 inhibitor HKI-272. Oncogene. 2007;26(34):5023–7. doi: 10.1038/sj.onc.1210292 17311002 10.1038/sj.onc.1210292

[9] Gilmer TM, Cable L, Alligood K, Rusnak D, Spehar G, Gallagher KT, et al Impact of common epidermal growth factor receptor and HER2 variants on receptor activity and inhibition by lapatinib. Cancer research. 2008;68(2):571–9. doi: 10.1158/0008-5472.CAN-07-2404 18199554 10.1158/0008-5472.CAN-07-2404

[10] Suzuki M, Shiraishi K, Yoshida A, Shimada Y, Suzuki K, Asamura H, et al HER2 gene mutations in non-small cell lung carcinomas: concurrence with Her2 gene amplification and Her2 protein expression and phosphorylation. Lung Cancer. 2015;87(1):14–22. doi: 10.1016/j.lungcan.2014.10.014 25468202 10.1016/j.lungcan.2014.10.014

[11] Sun Z, Shi Y, Shen Y, Cao L, Zhang W, Guan X. Analysis of different HER‐2 mutations in breast cancer progression and drug resistance. Journal of cellular and molecular medicine. 2015;19(12):2691–701. doi: 10.1111/jcmm.12662 26305917 10.1111/jcmm.12662 PMC4687700

[12] Slamon D, Clark G, Wong S, Levin W, Ullrich A, McGuire W. Human breast cancer: correlation of relapse and. science. 1987;3798106(177):235. 10.1126/science.3798106 3798106

[13] Slamon DJ, Godolphin W, Jones LA, Holt JA, Wong SG, Keith DE, et al Studies of the HER-2/neu proto-oncogene in human breast and ovarian cancer. science. 1989;244(4905):707–12. 2470152 10.1126/science.2470152

[14] Vogel CL, Cobleigh MA, Tripathy D, Gutheil JC, Harris LN, Fehrenbacher L, et al Efficacy and safety of trastuzumab as a single agent in first-line treatment of HER2-overexpressing metastatic breast cancer. Journal of Clinical Oncology. 2002;20(3):719–26. doi: 10.1200/JCO.2002.20.3.719 11821453 10.1200/JCO.2002.20.3.719

[15] Cobleigh MA, Vogel CL, Tripathy D, Robert NJ, Scholl S, Fehrenbacher L, et al Multinational study of the efficacy and safety of humanized anti-HER2 monoclonal antibody in women who have HER2-overexpressing metastatic breast cancer that has progressed after chemotherapy for metastatic disease. Journal of Clinical Oncology. 1999;17(9):2639–. doi: 10.1200/JCO.1999.17.9.2639 10561337 10.1200/JCO.1999.17.9.2639

[16] Seidman AD, Fornier MN, Esteva FJ, Tan L, Kaptain S, Bach A, et al Weekly trastuzumab and paclitaxel therapy for metastatic breast cancer with analysis of efficacy by HER2 immunophenotype and gene amplification. Journal of Clinical Oncology. 2001;19(10):2587–95. doi: 10.1200/JCO.2001.19.10.2587 11352950 10.1200/JCO.2001.19.10.2587

[17] MacFarlane RJ, Gelmon KA. Lapatinib for breast cancer: a review of the current literature. Expert opinion on drug safety. 2011;10(1):109–21. doi: 10.1517/14740338.2011.533168 21091041 10.1517/14740338.2011.533168

[18] Esteva FJ, Yu D, Hung M-C, Hortobagyi GN. Molecular predictors of response to trastuzumab and lapatinib in breast cancer. Nature reviews Clinical oncology. 2010;7(2):98–107. doi: 10.1038/nrclinonc.2009.216 20027191 10.1038/nrclinonc.2009.216

[19] Nahta R, Yu D, Hung M-C, Hortobagyi GN, Esteva FJ. Mechanisms of disease: understanding resistance to HER2-targeted therapy in human breast cancer. Nature clinical practice Oncology. 2006;3(5):269–80. doi: 10.1038/ncponc0509 16683005 10.1038/ncponc0509

[20] Wang Y-C, Morrison G, Gillihan R, Guo J, Ward RM, Fu X, et al Different mechanisms for resistance to trastuzumab versus lapatinib in HER2-positive breast cancers-role of estrogen receptor and HER2 reactivation. Breast Cancer Research. 2011;13(6):1. 10.1186/bcr3067 PMC3326563 22123186

[21] Zuo W-J, Jiang Y-Z, Wang Y-J, Xu X-E, Hu X, Liu G-Y, et al Dual characteristics of novel HER2 kinase domain mutations in response to HER2-targeted therapies in human breast cancer. Clinical Cancer Research. 2016;22(19):4859–69. doi: 10.1158/1078-0432.CCR-15-3036 27697991 10.1158/1078-0432.CCR-15-3036

[22] Verma S, Goyal S, Tyagi C, Jamal S, Singh A, Grover A. BIM (BCL-2 interacting mediator of cell death) SAHB (stabilized α helix of BCL2) not always convinces BAX (BCL-2-associated X protein) for apoptosis. Journal of Molecular Graphics and Modelling. 2016;67:94–101. doi: 10.1016/j.jmgm.2016.05.007 27262527 10.1016/j.jmgm.2016.05.007

[23] Nagpal N, Goyal S, Dhanjal JK, Ye L, Kaul SC, Wadhwa R, et al Molecular dynamics-based identification of novel natural mortalin–p53 abrogators as anticancer agents. Journal of Receptors and Signal Transduction. 2016:1–9. 10.3109/10799893.2016.1141952 27380217

[24] Pandey B, Grover S, Tyagi C, Goyal S, Jamal S, Singh A, et al Molecular principles behind pyrazinamide resistance due to mutations in panD gene in Mycobacterium tuberculosis. Gene. 2016;581(1):31–42. doi: 10.1016/j.gene.2016.01.024 26784657 10.1016/j.gene.2016.01.024

[25] Verma S, Grover S, Tyagi C, Goyal S, Jamal S, Singh A, et al Hydrophobic Interactions Are a Key to MDM2 Inhibition by Polyphenols as Revealed by Molecular Dynamics Simulations and MM/PBSA Free Energy Calculations. PloS one. 2016;11(2):e0149014 doi: 10.1371/journal.pone.0149014 26863418 10.1371/journal.pone.0149014 PMC4749206

[26] Sinha S, Tyagi C, Goyal S, Jamal S, Somvanshi P, Grover A. Fragment based G-QSAR and molecular dynamics based mechanistic simulations into hydroxamic-based HDAC inhibitors against spinocerebellar ataxia. Journal of Biomolecular Structure and Dynamics. 2016:1–15. 10.1080/07391102.2015.1113386 26510381

[27] Verma S, Tyagi C, Goyal S, Pandey B, Jamal S, Singh A, et al Mutations induce conformational changes in folliculin C-terminal domain: possible cause of loss of guanine exchange factor activity and Birt-Hogg-Dubé syndrome. Journal of Biomolecular Structure and Dynamics. 2016:1–6. 10.1080/07391102.2016.1188728 27484154

[28] Gupta A, Jamal S, Goyal S, Jain R, Wahi D, Grover A. Structural studies on molecular mechanisms of Nelfinavir resistance caused by non-active site mutation V77I in HIV-1 protease. BMC bioinformatics. 2015;16(19):1. 26695135 10.1186/1471-2105-16-S19-S10 PMC4686784

[29] Patel K, Tyagi C, Goyal S, Jamal S, Wahi D, Jain R, et al Identification of chebulinic acid as potent natural inhibitor of M. tuberculosis DNA gyrase and molecular insights into its binding mode of action. Computational biology and chemistry. 2015;59:37–47. doi: 10.1016/j.compbiolchem.2015.09.006 26410242 10.1016/j.compbiolchem.2015.09.006

[30] Gupta A, Jain R, Wahi D, Goyal S, Jamal S, Grover A. Abrogation of AuroraA-TPX2 by novel natural inhibitors: molecular dynamics-based mechanistic analysis. Journal of Receptors and Signal Transduction. 2015;35(6):626–33. doi: 10.3109/10799893.2015.1041645 26390942 10.3109/10799893.2015.1041645

[31] Kaburagi M, Yamada H, Miyakawa T, Morikawa R, Takasu M, Kato TA, et al Molecular dynamics simulation of telomeric single-stranded DNA and POT1. Polymer Journal. 2015;48:189 doi: 10.1038/pj.2015.82

[32] Berendsen HJ, van der Spoel D, van Drunen R. GROMACS: A message-passing parallel molecular dynamics implementation. Computer Physics Communications. 1995;91(1):43–56.

[33] Hess B, Kutzner C, Van Der Spoel D, Lindahl E. GROMACS 4: algorithms for highly efficient, load-balanced, and scalable molecular simulation. Journal of chemical theory and computation. 2008;4(3):435–47. doi: 10.1021/ct700301q 26620784 10.1021/ct700301q

[34] van Gunsteren WF, Billeter S, Eising A, Hünenberger PH, Krüger P, Mark AE, et al. Biomolecular simulation: The {GROMOS96} manual and user guide. 1996.

[35] Verma S, Singh A, Mishra A. Dual inhibition of chaperoning process by taxifolin: Molecular dynamics simulation study. Journal of Molecular Graphics and Modelling. 2012;37:27–38. doi: 10.1016/j.jmgm.2012.04.004 22609743 10.1016/j.jmgm.2012.04.004

[36] Pettersen EF, Goddard TD, Huang CC, Couch GS, Greenblatt DM, Meng EC, et al UCSF Chimera—a visualization system for exploratory research and analysis. Journal of computational chemistry. 2004;25(13):1605–12. doi: 10.1002/jcc.20084 15264254 10.1002/jcc.20084

[37] Kollman PA, Massova I, Reyes C, Kuhn B, Huo S, Chong L, et al Calculating structures and free energies of complex molecules: combining molecular mechanics and continuum models. Accounts of chemical research. 2000;33(12):889–97. 11123888 10.1021/ar000033j

[38] Kumari R, Kumar R, Lynn A. g_mmpbsa‐‐A GROMACS Tool for High-Throughput MM-PBSA Calculations. Journal of chemical information and modeling. 2014;54(7):1951–62. doi: 10.1021/ci500020m 24850022 10.1021/ci500020m

[39] Singh B, Bulusu G, Mitra A. Understanding the thermostability and activity of Bacillus subtilis lipase mutants: insights from molecular dynamics simulations. The Journal of Physical Chemistry B. 2015;119(2):392–409. doi: 10.1021/jp5079554 25495458 10.1021/jp5079554

[40] Shen H, Sun H, Li G. What is the role of motif D in the nucleotide incorporation catalyzed by the RNA-dependent RNA polymerase from poliovirus? PLoS Comput Biol. 2012;8(12):e1002851 doi: 10.1371/journal.pcbi.1002851 23300428 10.1371/journal.pcbi.1002851 PMC3531290

[41] Amadei A, Linssen A, Berendsen HJ. Essential dynamics of proteins. Proteins: Structure, Function, and Bioinformatics. 1993;17(4):412–25. 10.1002/prot.340170408 8108382

[42] Amadei A, Linssen A, De Groot B, Van Aalten D, Berendsen H. An efficient method for sampling the essential subspace of proteins. Journal of Biomolecular Structure and Dynamics. 1996;13(4):615–25. doi: 10.1080/07391102.1996.10508874 8906882 10.1080/07391102.1996.10508874

[43] Tournier AL, Smith JC. Principal components of the protein dynamical transition. Physical review letters. 2003;91(20):208106 doi: 10.1103/PhysRevLett.91.208106 14683404 10.1103/PhysRevLett.91.208106

[44] Van Aalten D, Findlay J, Amadei A, Berendsen H. Essential dynamics of the cellular retinol-binding protein evidence for ligand-induced conformational changes. Protein engineering. 1995;8(11):1129–35. 8819978 10.1093/protein/8.11.1129

[45] Wang L, Zeng R, Pang X, Gu Q, Tan W. The mechanisms of flavonoids inhibiting conformational transition of amyloid-[small beta]42 monomer: a comparative molecular dynamics simulation study. RSC Advances. 2015;5(81):66391–402. doi: 10.1039/c5ra12328c

[46] Bose R, Kavuri SM, Searleman AC, Shen W, Shen D, Koboldt DC, et al Activating HER2 mutations in HER2 gene amplification negative breast cancer. Cancer discovery. 2013;3(2):224–37. doi: 10.1158/2159-8290.CD-12-0349 23220880 10.1158/2159-8290.CD-12-0349 PMC3570596

[47] Trowe T, Boukouvala S, Calkins K, Cutler RE, Fong R, Funke R, et al EXEL-7647 inhibits mutant forms of ErbB2 associated with lapatinib resistance and neoplastic transformation. Clinical cancer research. 2008;14(8):2465–75. doi: 10.1158/1078-0432.CCR-07-4367 18413839 10.1158/1078-0432.CCR-07-4367

[48] Kancha RK, Von Bubnoff N, Bartosch N, Peschel C, Engh RA, Duyster J. Differential sensitivity of ERBB2 kinase domain mutations towards lapatinib. PloS one. 2011;6(10):e26760 doi: 10.1371/journal.pone.0026760 22046346 10.1371/journal.pone.0026760 PMC3203921

[49] Zabransky DJ, Yankaskas CL, Cochran RL, Wong HY, Croessmann S, Chu D, et al HER2 missense mutations have distinct effects on oncogenic signaling and migration. Proceedings of the National Academy of Sciences. 2015;112(45):E6205–E14. 10.1073/pnas.1516853112 PMC4653184 26508629

